# Are all Sedentary Behaviors Equal? An Examination of Sedentary Behavior and Associations with Indicators of Disease Risk Factors in Women

**DOI:** 10.3390/ijerph17082643

**Published:** 2020-04-12

**Authors:** Claire Beale, Erica L. Rauff, Wendy J. O’Brien, Sarah P. Shultz, Philip W. Fink, Rozanne Kruger

**Affiliations:** 1 School of Sport, Exercise, and Nutrition, Massey University, 4442 Palmerston North, New Zealand; claireb_1110@hotmail.com (C.B.); w.j.obrien@massey.ac.nz (W.J.O.); p.fink@massey.ac.nz (P.W.F.); r.kruger@massey.ac.nz (R.K.); 2 Kinesiology Department, Seattle University, Seattle, WA 98122, USA; rauffe@seattleu.edu

**Keywords:** sedentary behavior, accelerometry, disease risk factors

## Abstract

Sedentary behavior increases risk for non-communicable diseases; associations may differ within different contexts (e.g., leisure time, occupational). This study examined associations between different types of sedentary behavior and disease risk factors in women, using objectively measured accelerometer-derived sedentary data. A validation study (*n* = 20 women) classified sedentary behavior into four categories: lying down; sitting (non-active); sitting (active); standing. A cross-sectional study (*n* = 348 women) examined associations between these classifications and disease risk factors (body composition, metabolic, inflammatory, blood lipid variables). Participants spent an average of 7 h 42 min per day in sedentary behavior; 58% of that time was classified as non-active sitting and 26% as active sitting. Non-active sitting showed significant (*p* ≤ 0.001) positive correlations with BMI (r = 0.244), body fat percent (*r* = 0.216), body mass (*r* = 0.236), fat mass (*r* = 0.241), leptin (*r* = 0.237), and negative correlations with HDL-cholesterol (*r* = −0.117, *p* = 0.031). Conversely, active sitting was significantly (*p* ≤ 0.001) negatively correlated with BMI (*r* = −0.300), body fat percent (*r* = −0.249), body mass (*r* = −0.305), fat mass (*r* = −0.320), leptin (*r* = −0.259), and positively correlated with HDL-cholesterol (*r* = 0.115, *p* = 0.035). In summary, sedentary behavior can be stratified using objectively measured accelerometer-derived activity data. Subsequently, different types of sedentary behaviors may differentially influence disease risk factors. Public health initiatives should account for sedentary classifications when developing sedentary behavior recommendations.

## 1. Introduction

Sedentary behavior has been defined to include any behaviors (i.e., lying down or sitting stationary behaviors) that are ≤1.5 metabolic equivalents (METs) as well as passive standing (i.e., standing quietly in line, standing while cooking, sending text messages or talking on the phone while standing, reading while standing) that is ≤2.0 METs [1]. Estimates indicate that adults spend between 8 and 11 h (55–70%) of their waking day in sedentary behavior [2,3,4,5]. High levels of sedentary behavior have been linked with adverse health outcomes, independent of time spent in physical activity [6,7,8,9,10,11,12]. Increases in sedentary behavior due to the automation of transport, communications, workplace productivity, and entertainment [3] have occurred in conjunction with increased obesity rates [13,14]. Other non-communicable diseases associated with sedentary behavior, such as metabolic syndrome, type 2 diabetes, and cardiovascular disease [7,8,10,11,15,16,17] have also concurrently risen, suggesting a possible link between sedentary behavior and these diseases [18,19,20,21]. While levels of sedentary behavior and non-communicable diseases have been on the rise, the relationship between sedentary behavior and negative health outcomes appears to be complex.

The relationship between sedentary behavior and health outcomes is dependent on the types of sedentary activities and the age group under investigation [7]. For instance, strong evidence exists for the association between leisure time sedentary behavior and obesity in children and adolescents, but evidence is less clear in adults [7]. Furthermore, evidence supporting the association between negative health outcomes and occupational sitting specifically, is lacking. Some studies reported that sitting time at work was not associated with negative health outcomes [16,22,23]. For example, Picavet et al. [22] found that workers with sedentary jobs did not have an increased risk of negative health outcomes (i.e., hypertension, hypercholesterolemia) over 15 years, compared to those with non-sedentary jobs. This lack of relationship could be attributed to computer work contributing to a large proportion of activity performed while sitting in a “sedentary job” as research has found that typing while sitting exceeded the ≤1.5 MET threshold defining sedentary behavior [24]. Therefore, the small amount of increased energy expenditure associated with the seemingly sedentary activity of computer work may be sufficient to provide some degree of protection from chronic diseases, such as type 2 diabetes [23] and cardiovascular disease [25].

Incorporating light to moderate physical activity or postural changes by interrupting one’s sedentary behavior throughout the day may lead to beneficial changes in markers of disease risk factors [11,12,26,27,28,29,30,31,32]. Light to moderate physical activity, such as standing and walking slowly, can increase metabolic rate in comparison to more sedentary behaviors of sitting and lying [33], and thus provide positive effects on health indicators [34]. Subsequently, disruptions in sedentary time have also been associated with improvements in body composition [26,29,30]. Replacing sitting with light-intensity activities improves insulin action in healthy individuals [28] and in those suffering from type 2 diabetes [35]. Light intensity activities also decrease fasting triglycerides, total cholesterol, and non-high-density lipoprotein cholesterol while increasing high-density lipoprotein (HDL) cholesterol, apolipoprotein B, and non-esterified fatty acids [35,36], all of which affect mechanisms involved in cardiovascular disease and obesity. Based on these physiological differences, current trends in public health initiatives encourage changing work activities from sedentary to light to moderate physical activity (standing and light walking), with the goal of promoting accomplishable and sustainable changes in behavior [3,9]. Therefore, it is important to quantify sedentary behavior and light intensity activities, which can be measured using subjective or objective measures.

Subjective methods for assessing sedentary behavior involve questionnaires, activity diaries, or interviews where individuals report their activities or behaviors retrospectively [32,37,38]. Self-report of sedentary behaviors and physical activity through subjective methods can lead to overreporting of activity [39,40]. In contrast, objective methods of measuring sedentary behavior include accelerometers and inclinometers that are built into wearable devices, and these devices may be fitted to the waist, thigh, or wrist, as well as other body parts to measure body position and movement [41,42,43]. Objective methods of measuring sedentary behavior can be more precise and detailed than self-report methods [32].

Despite strong evidence for associations between sedentary behavior and disease risk factors, the associations in different contexts of sedentary behavior (e.g., occupational or leisure time) are contrasting [8,12,16,22,25]. Leisure time sedentary behavior is often associated with increased risk of disease [8,12], whereas little evidence exists for occupational sedentary behavior [16,22]. Contrasting associations between leisure and occupational sedentary behavior may particularly relate to sitting, since different energy expenditure estimates have been reported between typing and watching television while sitting [24]. Because there is variation in metabolic rates associated with differing sedentary postures, it is important to consider the effect of time spent in different sedentary behaviors on health outcomes. To date, there have been limited to no studies that have examined different categories of sedentary behavior and the effects these different categories of sedentary behavior have on disease risk factors. Thus, the purpose of this study was to examine the association between different types of low-MET sedentary behaviors (as previously defined by the Sedentary Behavior Research Network [1]) and indicators of disease risk factors in a sample of New Zealand women, using data from the women’s EXPLORE study [44].

## 2. Methods

The purpose statement was addressed across two studies. The first study was designed as a validation study (*n* = 20) to categorize posture and accelerometer counts, based on direct observation of various sedentary postures and activities. The second study applied the sedentary categories derived from Study One to accelerometer data collected during the women’s EXPLORE study [44].

### 2.1. Study One Methods

Twenty women (28 ± 9 years) were recruited in Auckland, New Zealand to participate in the validation study. Participants were included if they were post-menarche but pre-menopausal, and were excluded if they were pregnant, lactating, or diagnosed with a metabolic disorder. Ethical approval was obtained from Massey University Human Ethics Committee, and each participant gave written informed consent prior to participation. Participants were weighed without shoes and in minimal clothing to the nearest 0.1 kg using a digital scale (Seca, Birmingham, UK), and had their height measured to the nearest 0.1 cm using a stadiometer (Seca, Birmingham, UK).

A triaxial accelerometer (Actigraph wGT3X+, Actigraph Corp, Pensacola, FL, USA) was used to collect accelerometer/inclinometer data. A single accelerometer was fitted on the right hip (on the mid axillary line) of participants, using an adjustable elastic strap [33,45,46]. The hip-worn accelerometer was used to match the data collection protocol of the women’s EXPLORE study [44]. Participants followed a predetermined randomized sequence of body postures/activities, each of which was first demonstrated by the researcher. Participants were asked to maintain each posture/activity for five minutes. The researcher directly observed all postures and activities during validation. Accelerometer data were collected at a frequency of 100 Hz. A one-minute transition period between each activity was allowed. Descriptions of the postures/activities can be found in Table 1.

### 2.2. Study One Analyses

Raw accelerometer data were downloaded in 60-s epochs using ActiLife software (version 6.10.4, Actigraph Corp, Pensacola, FL, USA) and further analyzed in Matlab (R2013A, MathWorks, Natick, MA, USA). The Actilife software uses the offset angle measured by its inclinometer to identify counts per minute that are spent in standing (θ_y_ < 17 degrees), sitting (17 degrees < θ_y_ < 65 degrees), and lying (θ_y_ > 65 degrees) postures [47]. To avoid any issues due to changing positions, only the middle three minutes of each five-minute posture or activity were used. No differences in the activity counts were seen between postures, so classification was performed using the inclinometer counts per minute for lying, sitting, and standing. The inclinometer counts per minute for each 60-s epoch were plotted and grouped based on clustering. The clusters were confirmed by t-tests comparing the inclinometer counts, with a cluster being considered valid if no significant differences (*p* > 0.05) could be found between any of the postures or activities within the cluster. These clusters were then classified as lying, non-active sitting, active sitting, and standing (Table 1). The varying postures within each classification (e.g., standing vs. standing fidgeting within standing classification) did not differ in activity or inclinometer counts. Using this classification scheme, the classification matched the observation 84% of the minutes.

### 2.3. Study Two Methods

Detailed methods for this cross-sectional study have previously been published [45]. In summary, objectively measured physical activity data, body composition data, and markers of metabolic and cardiovascular health were collected as part of the women’s EXPLORE study. Ethical approval was gained from Massey University Human Ethics Committee. Written informed consent was obtained from each participant prior to data collection.

Participants included 406 women aged 16–45 years who identified with either Māori, Pacific, or New Zealand European ethnicities. Women were recruited in Auckland, New Zealand via media articles, advertising, flyers and posters in the local vicinity, as well as through social media, emailing lists from Massey University, and through face-to-face contact with community liaisons. Participants were included if they were post-menarche but pre-menopausal, and were excluded if they were pregnant, lactating, or diagnosed with a metabolic disorder; these criteria were assessed using a screening questionnaire.

A triaxial accelerometer (Actigraph wGT3X+, Actigraph Corp, Pensacola, FL, USA) was used to collect accelerometer/inclinometer data at 100 Hz during physical activity, sedentary behavior, and sleep, over a period of seven consecutive days. The accelerometer was fitted to the participant’s right hip on the mid axillary line, attached by an adjustable strap. Instructions were given to participants, directing them to wear the accelerometer at all times (excluding water-based activities). Sedentary behavior was defined as activities with <100 accelerometer counts per minute [48,49]. Participants were excluded from the current study if they did not meet the criteria for accelerometer wear time of ≥10 h per day on ≥4 week or weekend days. Non-wear time was determined by ≥60 continuous minutes of 0 counts per minute, with an allowance of 1–2 min of activity during that time. Valid data were received from 348 women; women with insufficient (*n* = 34) or no data (*n* = 24) were excluded.

### 2.4. Study Two Measures

*Demographics*: Demographic data were collected from participants via a questionnaire at the beginning of the study.

*Anthropometric and Biomarker Outcomes*: Detailed methodology of body composition measurements was published by Kruger et al. [44]. All anthropometric measurements were assessed with the participant in minimal and close-fitting clothing and without shoes. Waist and hip circumferences were measured to the nearest 0.1 cm using a metal Lufkin tape measure, according to the International Society for the Advancement of Kinanthropometry (ISAK) protocol [50]. Height was measured using a Harpenden stadiometer, to the nearest 0.1 cm. Body mass and body composition (body fat percentage and fat mass (kg)) were measured using air displacement plethysmography (BodPod, 2007A, Cosmed USA, Concord, CA, USA; manufacturer supplied software V4.2+). Body mass index (BMI; kg·m^−2^) was calculated using body mass and height.

Details of the full methodology used for metabolic testing is described elsewhere [44]. Briefly, a 26 mL fasting venous blood sample was taken from each participant in the morning to test metabolic, cardiovascular, and inflammatory markers. Blood was drawn into ethylene diamine tetra-acetic acid tubes to store 7 mL of plasma, 3.7 mL of serum, and an aliquot (0.5 mL) of whole blood was frozen at −80 °C for later analysis of glycated hemoglobin (HbA1c). HbA1c biomarkers were analyzed by Canterbury Scientific (Christchurch, New Zealand) using high performance liquid chromatography method (Bio-Rad Variant instrumentation, Bio-Rad Laboratories, Berkeley, CA, USA) [51]. The plasma and serum were centrifuged and used to measure the remaining biomarkers. Serum insulin was analyzed using immunoassay. An automated Dimension Vista (Siemens Healthcare Diagnostics) system analyzed C-reactive protein (CRP) (cat no K7032), cholesterol (cholesterol esterase and cholesterol oxidase method, cat no K1027), HDL-cholesterol (cholesterol oxidase and cholesterol esterase method, cat no K3048), triglyceride (lipase and glycerol kinase method, cat no K2069), and glucose (hexokinase and glucose-6-phosphate dehydrogenase method, cat no K1039). Low-density lipoprotein (LDL)-cholesterol was calculated using the Friedewald equation; total cholesterol to HDL-cholesterol ratio was also calculated. Plasma levels of interleukin-6 (IL-6), interleukin-10 (IL-10), tumor necrosis factor alpha (TNF-a), ghrelin, and leptin were measured using Milliplex immunoassay kits (Millipore Corp., Billerica, MD, USA). Blood pressure was measured three times with one-minute rest between measurements using an automated blood pressure monitor (Riester Ri Champion, Rudolf Riester GmbH, Jungingen, Germany).

*Total Daily Energy Intake*: Total daily energy intake was used as a covariate for markers of overweight and obesity in the analysis. Participants completed an online one-month food frequency questionnaire [52]. Total daily energy intake was calculated using data from the questionnaire and analyzed through FoodWorks dietary analysis software (FoodWorks Professional 7; Xyris Software, Australia; New Zealand Food Composition Database).

### 2.5. Study Two Analyses

Actigraph data were downloaded at 60-s epochs using ActiLife software (version 6.7.1, Actigraph Corp, Pensacola, FL, USA). Sedentary behavior classification codes identified in the validation study were applied to accelerometer data on a minute-by-minute basis using Matlab (R2013A, MathWorks, Natick, MA, USA). Each minute was first classified as either sleep, non-wear, or awake, using the method in Barreira et al. [53]. All awake minutes <100 counts per minute were classified as sedentary [54], and were further sorted into categories of lying, non-active sitting, active sitting, or standing, based on the inclinometer criteria obtained from the validation study. Following this classification, the data were sorted into individual days to find the total amount of sedentary time for each day, as well as the total amount of time per day for each of the four classifications of sedentary behavior.

Statistical analyses were carried out using SPSS Statistics 22 for Windows (SPSS, Inc., Chicago, IL, USA). A one-way ANOVA with post-hoc paired 2-tailed t-tests was performed to analyze the differences between time spent in the different categories of sedentary behavior; Bonferroni correction was completed with an alpha level of *p* < 0.05/4 = 0.0125. In order to understand the relationship between different types of sedentary behavior and disease risk factors indicators, bivariate correlations (Pearson’s) were performed for sedentary categories and all body composition and biomarker variables.

Sedentary time (total and by sub-category) was an average of the recorded sedentary time (min) per valid day. Data are presented as mean (± standard deviation) and correlation coefficients (significance). Significance for statistical analysis was set at *p* < 0.05 for all variables, unless otherwise noted.

## 3. Results

Participants were 348 women (Māori, *n* = 68; Pacific, *n* = 65; New Zealand European, *n* = 215) with an average age of 32.6 ± 8.5 years. BMI range was 18.7–49.1 kg·m^−2^, and prevalence of overweight was 54.6%. Further participant characteristics are presented in Table 2.

Results of the classification scheme applied to the accelerometer data are shown in Table 3. On average, participants spent 7 h and 42 min (±1 h and 12 min) engaging in sedentary behavior during one waking day. Of this time, the majority was spent in the non-active sitting classification (58%), followed by active sitting (26%). The remainder of the sedentary time was spent lying (9%) or standing (7%). There was a significant difference (*p* ≤ 0.001) in the amount of time spent in the four different types of sedentary behavior.

There were no correlations between total sedentary behavior and any body composition variables or markers of metabolic disease (Table 4). Total sedentary behavior time was positively correlated with systolic blood pressure, but with no other cardiovascular disease markers.

Lying and non-active sitting postures followed similar directional patterns of weak correlations with body composition variables, metabolic markers. and cardiovascular markers (Table 4). Likewise, active sitting and standing postures followed similar directional patterns of weak to moderate correlations with body composition variables, metabolic markers, and cardiovascular markers (Table 4). Correlational analysis did not produce any statistically significant associations between sedentary postures and some cardiometabolic (serum glucose, HbA1c, LDL-cholesterol) and inflammatory (IL-6, IL-10, TNF-α) markers. Thus, these variables have not been included in Table 4.

## 4. Discussion

In the current study, we initially developed a specific classification algorithm using the dataset from the validation study we conducted. The algorithm is a valuable tool that identified four categories of sedentary behaviors based on differences in inclinometer data. The classification algorithm provided 84% agreement between observed behaviors and accelerometer data and is comparable in accuracy to previous studies [5,55,56]. After cross-sectional accelerometer data were reclassified using the algorithm, bivariate correlations analyzed the implications of different types of sedentary behaviors on disease risk factors in a sample of New Zealand women. In particular, active sitting (i.e., within the limits of sedentary behavior, <100 counts per minute) was inversely associated with markers of overweight and obesity, metabolic disorders, and cardiovascular disease risk factors, and such findings warrant further discussion.

Total sedentary time in the current study was not associated with overweight/obesity, or with any markers of metabolic or cardiovascular disease risk factors, with the exception of a positive correlation with systolic blood pressure. Conversely, many previous studies found associations between sedentary behavior and markers of cardiovascular [57,58,59,60] or metabolic disease risk factors [15]. Differing sedentary behavior measurements could be the mechanism producing these conflicting results between previous work and the current study. While the previous studies used self-reported total sitting time to estimate total sedentary behavior, the current study objectively measured all sedentary behaviors. The Whitehall II study [61] demonstrated this balance between positive and negative effects of sedentary behavior when it found that prior obesity was related specifically to time spent watching television, but not to other types of sitting, and not to total sitting time. The previously reported effects of total sedentary behavior on markers of disease risk factors are likely dependent on the specific type of sedentary behavior that contributed most to total sedentary time, a factor that cannot be determined from most previous studies.

Unlike total sedentary behavior, the categories of sedentary behavior presented in the current study had clear differing associations with health and disease risk factors. The major finding was that active sitting was inversely associated with disease risk factors. Active sitting appears to be most representative of occupational sitting time (e.g., working on a computer at a desk and/or typing), as opposed to leisure time sitting behaviors, such as watching television. This difference is likely due to the active sitting classification category used in the current study, which included sitting typing and sitting on a stool, but not sitting still or reclining (more common when sitting watching television). These findings are similar to previous cohort research [25], which reported a lower risk of mortality in women who fidget while sitting compared to those who do not. However, in other studies no association between occupational sitting and markers of cardiometabolic health [22] and obesity [23,61] have been reported. In particular, Gupta et al. [4] found that long bouts of sitting at work, but not during leisure time, were positively correlated with obesity. However, Gupta et al. [4] categorized sitting time into occupational and leisure time, both of which could include a variety of non-active and active sedentary behaviors. When identified by activity levels and not location of the sedentary behavior, it would seem that a more active sedentary task (e.g., sitting typing) is associated with reduced disease risk factors, relative to performing a less active sedentary task (e.g., television watching).

Given the association between non-active sitting and unfavorable markers of disease risk factors that is supported by numerous studies [8,23,58,59,61,62,63,64,65], it is unsurprising that non-active sitting was associated with an increase in disease risk factors in the current study. Although previous studies have not defined types of sedentary behavior in the same way as the current study, non-active sitting can be considered most closely related to sitting watching television. Increased television viewing time has been associated in multiple studies with increased cardiovascular disease risk factors [58,59,63,64] and with metabolic disease risk factors in young women [65]. Furthermore, individuals with prior obesity were found to spend more time watching television than those of normal weight [61]. Overall, increased television viewing time is associated with negative health implications in the majority of research, and strategies to reduce this inactive sitting behavior present a simple target for public health messaging.

This study is unique in that sedentary behavior was classified according to both posture and activity type using objectively measured data, and these classifications were further used to establish associations between different types of sedentary behavior and disease risk factors. However, the current study has a number of limitations that must be acknowledged. The data from this study were collected in a sample of relatively young, healthy females who did not report current cardiometabolic disease. Future work should examine these associations in other populations (i.e., older adults, males, diseased) to understand if these associations are present in more diverse samples. The objective data was not accompanied by subjective data to provide context to the various types of sedentary behaviors; however, the use of categories to classify sedentary behaviors may negate the need for such contextual understanding. The study was conducted in New Zealand women between the ages of 16 and 45 years, so findings may not be generalizable to some other population groups. As with any cross-sectional data, which provides a snapshot of the population in time, causation cannot be determined. Longitudinal studies of longer duration may be needed to better understand if individuals who engage in higher levels of active sitting actually have decreased negative health outcomes when compared to those who tend to engage in more inactive sitting. Further investigation of objectively measured sedentary behavior could identify the long-term effects of different types of sedentary behavior on disease risk factors and disease outcome, in order to reduce the burden of disease associated with excessive inactive sedentary behaviors.

## 5. Conclusions

The current study highlights an interesting association, where objectively measured sedentary behavior, with MET values towards the upper end of the sedentary range, might have positive effects on markers of non-communicable disease risk factors. Taking the current findings into consideration, it may be useful to consider public health messaging that focuses on changes people can make to their sedentary behavior, such as encouragement of active sitting to potentially reduce the population risk of non-communicable diseases. Active sitting made up only 26% of total sedentary behavior. Therefore, messages that suggest ways to reallocate sedentary time from ‘inactive’ to ‘active’ sedentary behaviors may be one mechanism for reducing the increase in prevalence of non-communicable diseases associated with excessive sitting.

## Figures and Tables

**Table 1:** Classification of sedentary postures/activities into four categories.

Category of Posture	Inclinometer-Lying	Inclinometer-Sitting	Inclinometer-Standing	Posture/Activity
Lying	≥10 cpm			1. Lying supine
				2. Lying supine, with knees bent
				3. Lying right lateral side
Non-active sitting	<10 cpm		<10 cpm	1. Lying left lateral side *
				2. Reclining
				3. Sitting on a chair (with backrest and feet on the floor)
				4. Sitting, right leg crossed over the left knee
				5. Sitting, left leg crossed over the right knee
Active sitting	<10 cpm	<30 cpm	≥10 cpm	1. Sitting on a stool (without backrest and feet off the floor)
				2. Sitting, typing on a keyboard
Standing (cpm)	<10 cpm	≥30 cpm	≥10 cpm	1. Standing
				2. Standing, fidgeting with paper

cpm: Counts per minute; * (lying left lateral side classified as non-active sitting because the posture/activity could not be differentiated from other postures/activities within the non-active sitting group based on accelerometer data).

**Table 2:** Participant anthropometric and biomarker characteristics (*n* = 348).

Variable	Mean	SD
Total energy intake (kJ)	9686.5	3417.3
*Anthropometric variables*		
BMI (kg·m^−2^)	27.0	5.7
Body mass (kg)	75.1	16.3
Fat mass (kg)	25.3	9.0
Body fat (%)	33.4	8.0
Waist:Hip Ratio	0.8	0.1
*Biomarker variables*		
Insulin (mmol·L^−1^)	11.7	8.3
Serum glucose (mmol·L^−1^)	4.7	0.4
HbA1c (mmol·mol^−1^)	28.6	3.7
CRP (nmol·L^−1^)	3.9	3.2
IL-6 (pg·mL^−1^)	2.4	1.8
IL-10 (pg·mL ^−1^)	14.8	12.9
TNF-α (pg·mL^−1^)	6.7	2.5
Leptin (μg·mL^−1^)	101.1	7.0
Ghrelin (pg·mL^−1^)	48.0	39.9
Systolic blood pressure (mmHg)	116.3	10.0
Diastolic blood pressure (mmHg)	73.0	8.3
Cholesterol (mmol·L^−1^)	4.6	0.9
HDL-cholesterol (mmol·L^−1^)	1.6	0.4
Triglycerides (mmol·L^−1^)	1.0	0.7
TC:HDL	3.1	0.9
LDL-cholesterol (mmol·L^−1^)	2.6	0.8

Abbreviations: BMI, body mass index; HbA1c, glycated hemoglobin; CRP, C-reactive protein; IL-6, interleukin 6; IL-10, interleukin 10; TNF-a, tumor necrosis factor alpha; HDL, high density lipoprotein; TC:HDL, total cholesterol to high density lipoprotein ratio; LDL, low density lipoprotein. SD, standard deviation.

**Table 3:** Time spent in different types of sedentary behavior.

Accelerometer Derived Variables (min/day)	Mean	SD
**Total sedentary time**	**464.2**	**72.8**
Lying time	43.4	31.1
Sitting time non-active	269.0	75.9
Sitting time active	120.9	63.1
Standing time	30.8	12.5

Note. All classifications were significantly different from the other types of sedentary behavior (*p* ≤ 0.001).

**Table 4:** Pearson’s correlations of sedentary behavior with anthropometric variables and biomarkers.

Variable	Lying	Non-Active Sitting	Active Sitting	Standing
Age (years)	−0.187 ^†^	NS	NS	−0.120 *
Total energy intake (kJ)	0.167 ^†^	−0.111 *	NS	NS
*Body composition*				
BMI (kg·m^−2^)	0.232 ^†^	0.244 ^†^	−0.300 ^†^	−0.285 ^†^
Body mass (kg)	0.252 ^†^	0.236 ^†^	−0.305 ^†^	−0.277 ^†^
Body fat (%)	0.111 *	0.216 ^†^	−0.249 ^†^	−0.290 ^†^
Fat mass (kg)	0.245 ^†^	0.241 ^†^	−0.323 ^†^	−0.320 ^†^
Waist:Hip	NS	0.141 ^#^	−0.164 ^†^	−0.258 ^†^
*Cardiometabolic markers*				
Insulin (mmol·L^−1^)	0.173 ^†^	0.160 ^†^	−0.180 ^†^	−0.124 *
CRP (nmol·L^−1^)	NS	NS	NS	−0.120 *
Leptin (ng·mL^−1^)	0.185 ^†^	0.237 ^†^	−0.259 ^†^	−0.267 ^†^
Ghrelin (pg·mL^−1^)	−0.166 ^†^	NS	NS	NS
HDL−cholesterol (mmol·L^−1^)	NS	−0.117 *	0.115 *	0.147 ^#^
Triglycerides (mmol·L^−1^)	0.142 ^#^	−0.005	NS	−0.150 ^#^
TC: HDL	NS	NS	NS	−0.153 ^#^
*Blood Pressure*				
Systolic blood pressure (mmHg)	0.137 *	0.137 *	NS	NS
Diastolic blood pressure (mmHg)	0.120 *	0.135 *	NS	NS

Values are Pearson’s correlation coefficients. Abbreviations: NS, Non-Significant; SB, sedentary behavior; BMI, body mass index; HbA1c, glycated hemoglobin; CRP, C-reactive protein; IL-6, interleukin 6; IL-10, interleukin 10; TNF-a, tumor necrosis factor alpha; HDL, high density lipoprotein; TC:HDL, total cholesterol to high density lipoprotein ratio; LDL, low density lipoprotein. Significant correlations are denoted by the following: * (*p* < 0.05); ^#^ (*p* < 0.01); ^†^ (*p* < 0.005).
